# A novel SNP in the 5’ regulatory region of organic anion transporter 1 is associated with chronic kidney disease

**DOI:** 10.1038/s41598-018-26460-y

**Published:** 2018-05-24

**Authors:** Chiao-Yin Sun, Mai-Szu Wu, Chin-Chan Lee, Shu-Hong Chen, Kang-Chieh Lo, Yau-Hung Chen

**Affiliations:** 1 Department of Nephrology, Keelung Chang Gung Memorial Hospital, Keelung, Taiwan; 2 College of Medicine, Chang Gung University, Taoyuan, Taiwan; 3 Department of Internal Medicine, School of Medicine, College of Medicine, Taipei Medical University, Taipei, Taiwan; 4 Division of Nephrology, Department of Internal Medicine, Taipei Medical University Hospital, Taipei, Taiwan; 5 Medical Research Center, Keelung Chang Gung Memorial Hospital, Keelung, Taiwan; 6 Department of Chemistry, Tamkang University, Tamsui New Taipei City, Taiwan

## Abstract

We aimed to analyze the associations of single nucleotide polymorphisms (SNP) in the 5′ regulatory region of the human organic anion transporter 1 (OAT1) gene with chronic kidney disease (CKD). A case-control study including age- and sex-matched groups of normal subjects and patients with CKD (n = 162 each) was designed. Direct sequencing of the 5′ regulatory region (+88 to −1196 region) showed that patients with CKD had a higher frequency of the −475 SNP (T > T/G) than normal subjects (14/162 *vs*. 2/162). The luciferase activity assay results indicated that the −475G SNP had a higher promoter efficiency than the −475T SNP. Chromatin immunoprecipitation (ChIP) and LC/MS/MS analyses showed that the −475G SNP up-regulated 26 proteins and down-regulated 74 proteins. The Southwestern blot assay results revealed that the −475G SNP decreased the binding of Hepatoma-derived growth factor (HDGF), a transcription repressor, compared to the −475T SNP. Overexpression of HDGF significantly down-regulated OAT1 in renal tubular cells. Moreover, a zebrafish animal model showed that HDGF-knockdown zebrafish embryos had higher rates of kidney malformation than wild-type controls [18/78 (23.1%) vs. 1/30 (3.3%)]. In conclusion, our results suggest that an OAT1 SNP might be clinically associated with CKD. Renal tubular cells with the −475 SNP had increased OAT1 expression, which resulted in increased transportation of organic anion toxins into cells. Cellular accumulation of organic anion toxins caused cytotoxicity and resulted in CKD.

## Introduction

The pathological course of chronic kidney disease (CKD) forms a virtuous circle. Current studies have revealed that anionic uremic toxins, such as indoxyl sulfate (IS) and p-cresol sulfate (PCS), can increase oxidative stress and induce cell apoptosis. Clinical evidence has also shown that decreasing IS and PCS through treatment with an oral spherical carbon adsorbent can protect against deterioration of renal function in patients with CKD^1^.

The family of organic anion transporters (OATs) belongs to the major facilitator superfamily (SLC22A), and OATs are expressed in renal tubular epithelial cells to regulate excretion and reabsorption of endogenous and exogenous organic anions, including drugs and their metabolites^2^. Recently, it was revealed that anionic uremic toxins are physiological substrates for the OAT family and that their accumulation within renal tubules through the activity of OATs induces renal dysfunction^3–5^. For example, OAT1, a prototypical OAT, is reported to play a central role in the renal secretion of organic anions. OAT1 mediates the uptake of a wide range of relatively small and hydrophilic organic anions from plasma into the cytoplasm of proximal tubular cells, allowing these organic anion toxins to then be pumped out by other types of OATs^6^. Substantial evidence indicates that OAT1 plays a critical role in kidney injury by mediating accumulation of organic anionic toxins in the kidney^7–9^. Renal clearance of organic anions varies among individuals^10^. Clinically, the serum levels of IS and PCS, organic solutes transported into renal tubular cells by OAT1, have been shown to vary among subjects with similar renal function^11–13^, suggesting that genetic factors might contribute to interindividual differences in renal clearance of organic anionic toxins.

The genes encoding OAT1 and other solute transporters are clustered on human chromosome 11, with OAT1 and OAT3 genes existing on chromosome 11 as a tandem pair. Previous studies have demonstrated that the selective pressure on the coding sequences of OAT1 is relatively small compared with that of other OAT family members^14^, suggesting that OAT1 is a conserved protein. Clinical studies have also indicated that OAT1 has low genetic and functional diversity in coding regions^15^. Therefore, this study aimed to analyze single nucleotide polymorphisms (SNPs) in the 5′ regulatory region of human OAT1 (SLC22A6) and their possible clinical associations with CKD. We screened for variants in the 5′ regulatory region of OAT1 in DNA samples from normal subjects and subjects with CKD (n = 162 for each group), and the associations between regulatory polymorphisms and CKD were analyzed. We also performed cellular studies to investigate the molecular mechanism of expression regulated by polymorphisms in the 5′ regulatory region of human OAT1.

## Results

**Figure 1:**
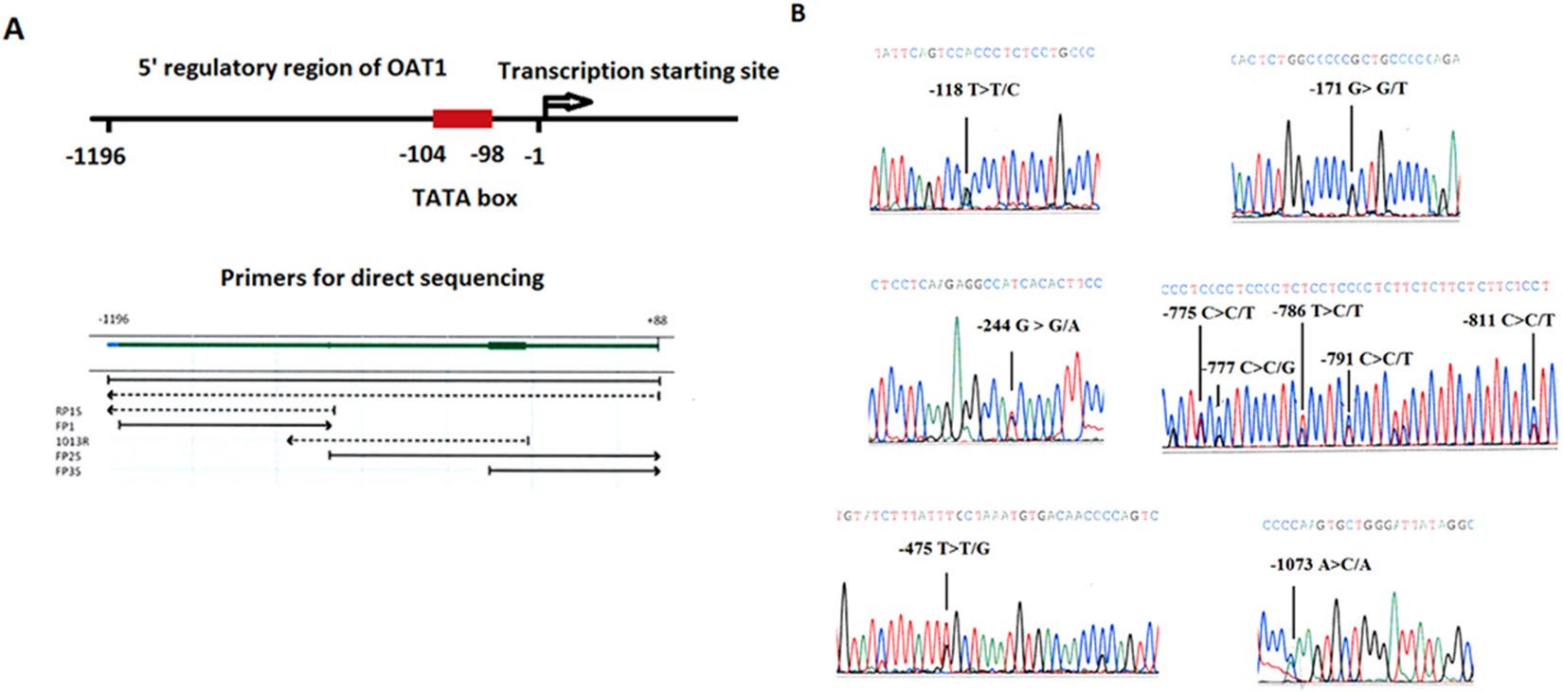
Direct sequencing of the OAT1 5′ regulatory region. (**A**) The predicted TATA box of the promoter was located at positions −98 to −104 from the transcription start site. Polymorphisms of the 5′ regulatory region of OAT1 from +88 to −1196 bp were analyzed by direct sequencing. The locations and directions of the direct sequencing primers are illustrated in the figure. (**B**) Chromosomal DNA from peripheral leukocytes was analyzed by direct sequencing. There were 10 SNPs on the 5′ regulatory region of OAT1. The representative graphs for these SNPs are displayed.

The reference sequence of the 5′ regulatory region of human OAT1 from +88 to −1196 bp and the results of promoter prediction are shown in Supplemental Fig. S1. There were 3 potential promoter regions predicted on the 5′ regulatory region of OAT1 from −1 to −1196 bp. To define possible clinical associations between the SNPs of OAT1 and CKD, DNA fragments containing the 5′ regulatory region of OAT1 (from +88 to −1196 bp) were analyzed with chromosomal DNA of peripheral leukocytes by direct sequencing (Fig. 1A). The study included 324 study subjects divided into age- and sex-matched normal (n = 162) and CKD (n = 162) groups. The characteristics of study subjects are listed in Table 1. Ten SNPs were found by direct sequencing, and the frequencies of these SNPs for each group are summarized in Table 2. Four of the SNPs found in this study (−118, −171, −791, −811) have been previously identified and assigned an rs number in the SNP database (dbSNP). Six of the SNPs found in this study (−244, −475, −775, −777, −786, −1073) are new variants, and no corresponding rs numbers were found (Table 2). The representative sequencing results are shown in Fig. 1B. The −475 SNP with the change of T > T/G was the most common SNP in the study population (16/324, 4.9%). In addition, the frequency of the −475 SNP in subjects with CKD was significantly higher than in normal subjects (14/162 *vs*. 2/162; P = 0.003). The frequencies of other SNPs did not differ significantly between normal (control) and CKD groups (Table 2). These results suggest that the −475 SNP (T > T/G) of OAT1 is clinically associated with CKD. The odds ratio for subjects with the −475 SNP (T > T/G) having CKD was 7.57 (95% Confidence Interval: 1.69–33.86; P = 0.008).

**Table 1:** Characteristics of study subjects and results of direct sequencing.

	Normal control n = 162	CKD n = 162
Age (y/o)	64.15 ± 13.01	64.74 ± 12.29
Gender (F/M)	81/81	81/81
eGFR	96.48 ± 11.27	32.02 ± 14.78*
**CKD stage**		
3		n = 72
4		n = 35
5		n = 55

(A) the characteristics of study subjects. The frequency of −475 polymorphism (T > T/G) of CKD subjects was significantly higher than normal subjects (P = 0.003). ^*^P < 0.05.

**Table 2:** The results of polymorphism frequency of study subjects by direct sequencing.

SNP Location	SNP	rs number	Normal	CKD
			control	(n = 162)
			(n = 162)	
−118 (62985115)	T > T/C	rs58244957	0	1
−171 (62985168)	G > G/T	rs57350702	0	1
−244 (62985241)	G > G/A		1	1
−475 (62985472)	T > T/G		2	14*
−775 (62985772)	C > C/T		1	2
−777 (62985774)	C > C/G		0	2
−786 (62985783)	T > C/T		1	2
−791 (62985788)	C > C/T	rs978103765	1	2
−811 (62985808)	C > C/T	rs1014912263	0	1
−1073 (62986070)	A > C/A		2	0

^()^Location of human chromosome 11.

^*^P = 0.003, Fisher’s exact test.

**Figure 2:**
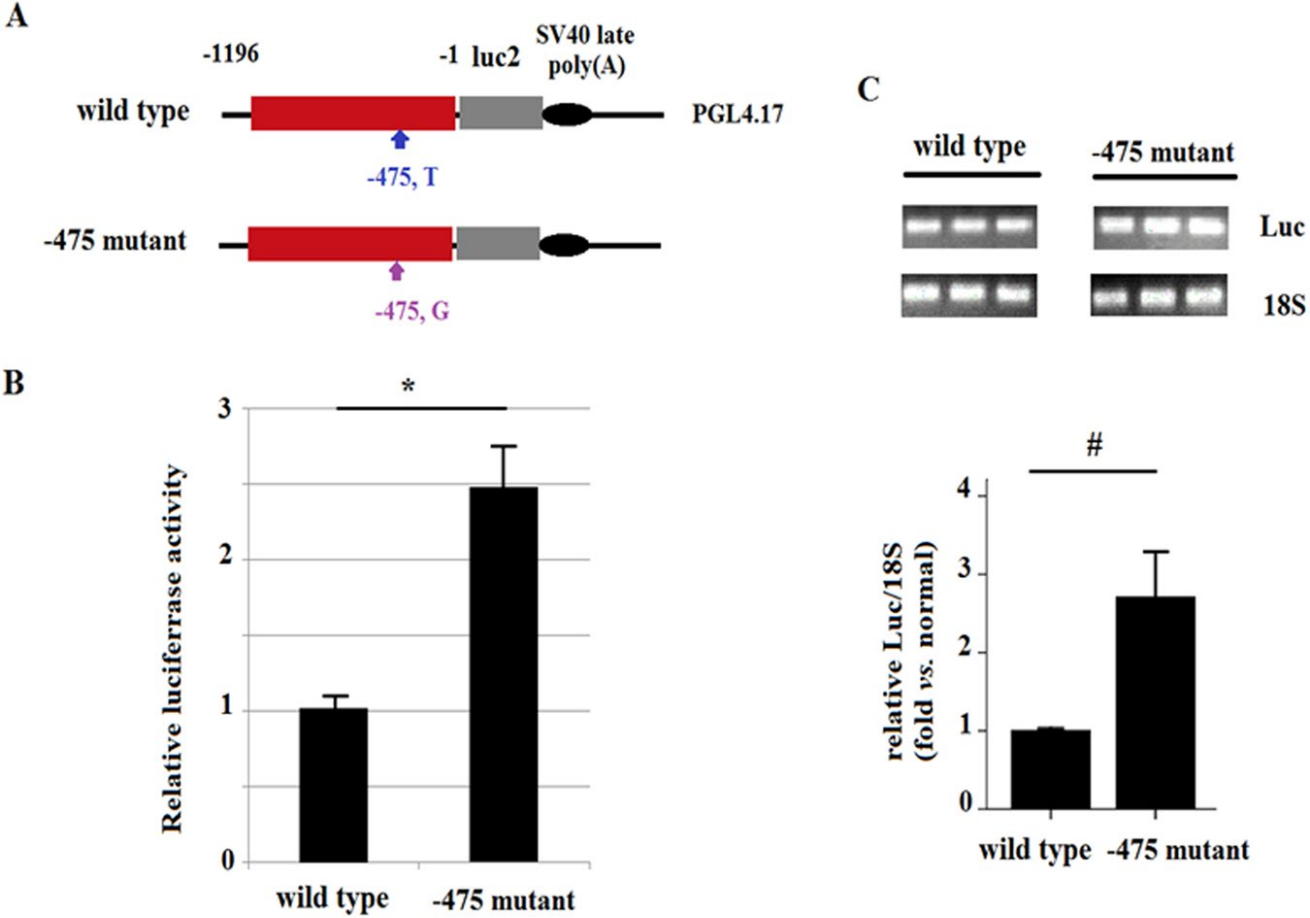
Promoter efficiency analysis by luciferase activity assay. (**A**) Plasmids for luciferase activity were constructed by inserting a synthetic 5′ regulatory region of OAT1 (−1 to −1196) into the pGL4.17 vector. The wild-type and −475 mutant plasmids differed only in the nucleotide at the −475 position. (**B**) The results of the luciferase activity assay are shown. Cell lysates of HK2 cells transfected with reporter plasmids were harvested for luciferase activity analysis. (**C**) The results of real-time PCR for luciferase and 18S RNA. The RNA samples from HK2 cells transfected with reporter plasmids were analyzed by real-time PCR, and the PCR products were analyzed semi-quantitatively by electrophoresis. Each reaction for (**B**) and (**C**) was repeated in triplicate. (*P = 0.019; ^#^P = 0.003).

**Figure 3:**
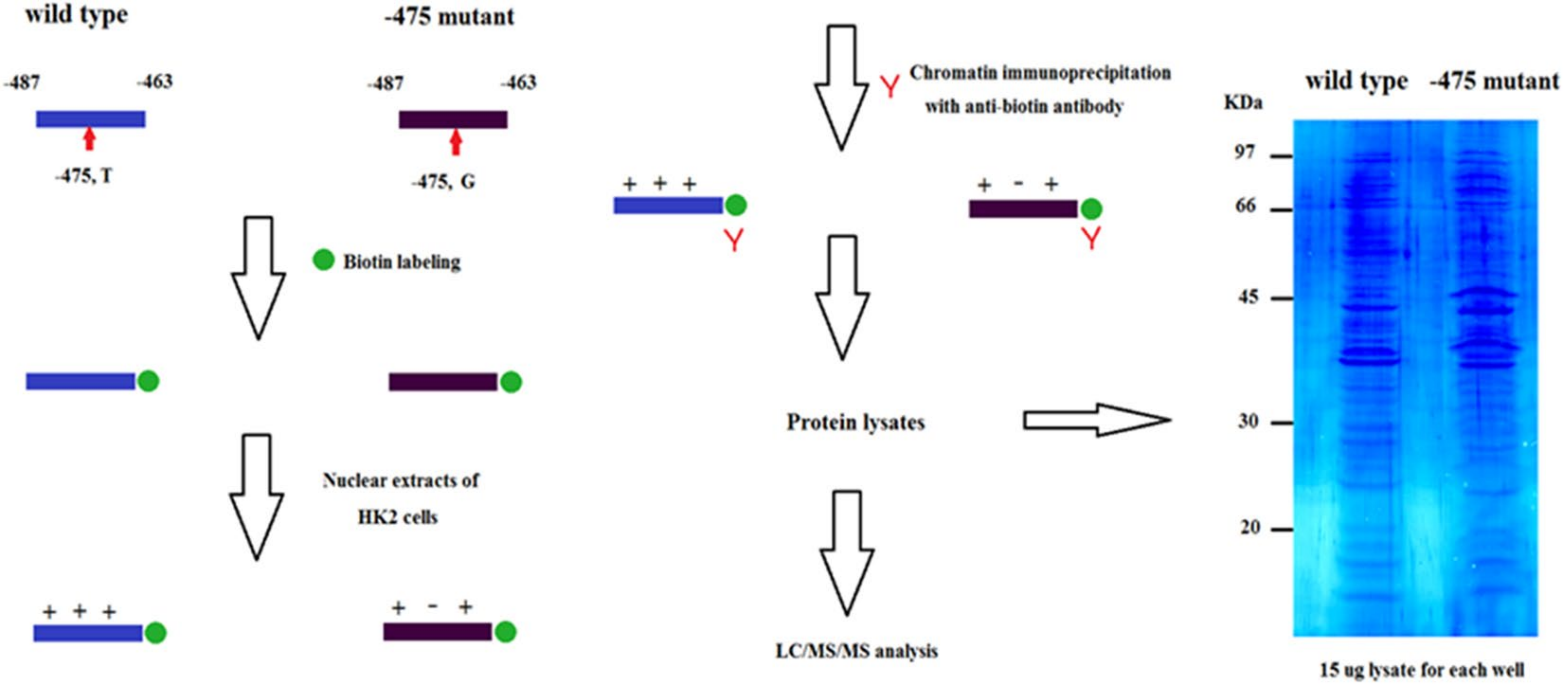
Chromatin immunoprecipitation analysis flow. Biotin-labeled synthetic oligonucleotides (−463 to −487) with or without the −475 mutation (T to G) were incubated with HK2 nuclear extracts. Chromatin immunoprecipitation was performed with an anti-biotin antibody. The *Coomassie blue-stained* immunoprecipitated protein lysates are shown. The immunoprecipitated protein lysates were further subjected to LC/MS/MS analysis.

To elucidate possible effects of the −475 SNP on expression of OAT1, a promoter activity assay with wild-type and −475 mutant promoters (−1 to −1196 nt) was performed (Fig. 2A). The −475 mutant promoter increased luciferase activity relative to the wild-type promoter (2.5 *vs*. 1.0; P = 0.019) (Fig. 2B). Real-time PCR results also showed that the −475 mutant promoter increased luciferase mRNA expression relative to the mutant promoter (P = 0.003) (Fig. 2C). These results indicate that the −475 SNP of OAT1 might up-regulate OAT1 expression by affecting transcription factor binding. A chromatin immunoprecipitation/LC/MS/MS analysis using wild-type and −475 mutant oligonucleotides (−463 to −487; 25 bp) was conducted to identify potential binding transcription factors. The chromatin immunoprecipitation analysis flow is summarized in Fig. 3. Compared with the wild-type oligonucleotide, 26 proteins were up-regulated and 74 proteins were down-regulated (2-fold changes, 2 peptides identified) by the −475 mutant oligonucleotide. Functional ontology analysis showed that 17 of the proteins with altered expression were classified under transcription regulation (Table 3).

**Table 3:** Results of LC/MS/MS analysis.

Protein-ID	Protein Name	Mass(Da)	pI	Fold change
Q13185	Chromobox protein homolog 3 < sp|Q13185|CBX3_HUMAN>	20811.42	5.23	−100
Q06710	Paired box protein Pax-8 < sp|Q06710|PAX8_HUMAN>	48217.73	7.72	−100
P51858	Hepatoma-derived growth factor < sp|P51858|HDGF_HUMAN>	26788.29	4.7	−100
O60869	Endothelial differentiation-related factor 1 < sp|O60869|EDF1_HUMAN>	16237.49	9.95	−100
Q9Y330	Zinc finger and BTB domain-containing protein 12 < sp|Q9Y330|ZBT12_HUMAN>	49147.5	7.26	−100
Q32MQ0	Zinc finger protein 750 < sp|Q32MQ0|ZN750_HUMAN>	77360.57	8.45	−100
P42568	Protein AF-9 < sp|P42568|AF9_HUMAN>	63351.38	8.77	−100
P82979	SAP domain-containing ribonucleoprotein < sp|P82979|SARNP_HUMAN>	23539.62	6.12	−100
P16402	Histone H1.3 < sp|P16402|H13_HUMAN>	22218.71	11.02	−100
P04908	Histone H2A type 1-B/E < sp|P04908|H2A1B_HUMAN>	14004.3	11.05	−3.58
Q96I24	Far upstream element-binding protein 3 < sp|Q96I24|FUBP3_HUMAN>	61509.23	8.61	−2.348
P18754	Regulator of chromosome condensation < sp|P18754|RCC1_HUMAN>	44837.83	7.17	−2.238
O15446	DNA-directed RNA polymerase I subunit RPA34 < sp|O15446|RPA34_HUMAN>	54985.6	8.66	100
Q96IZ0	PRKC apoptosis WT1 regulator protein < sp|Q96IZ0|PAWR_HUMAN>	36567.51	5.35	100
O14776	Transcription elongation regulator 1 < sp|O14776|TCRG1_HUMAN>	123901.03	8.71	100
Q9UIF8	Bromodomain adjacent to zinc finger domain protein 2B < sp|Q9UIF8|BAZ2B_HUMAN>	240459.17	6.13	100
Q6P1N0	Coiled-coil and C2 domain-containing protein 1 A < sp|Q6P1N0|C2D1A_HUMAN>	104062.48	8.22	100

There were 26 proteins up-regulated and 74 proteins down-regulated by −475 mutants. Among these targets, 17 proteins were functionally classified as transcription regulation. Those transcription regulation proteins were listed. (Fold change: −475 mutant *vs*. wild type).

**Figure 4:**
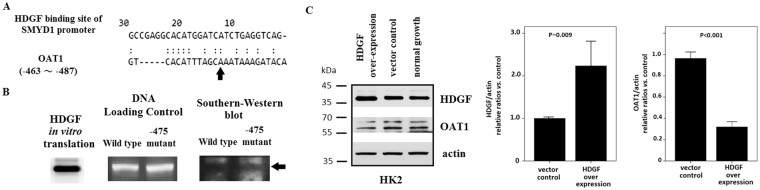
HDGF down-regulated OAT1 expression. (**A**) Alignment analysis of the HDGF binding site of the SMYD1 promoter (30 bp) with the 5′ regulatory region of OAT1 (−463 to −487, 25 bp) revealed 43.3% sequence identity over a 30 nt overlap. The nucleotide at the −475 position of the OAT1 promoter was identical to that of the SMYD1 promoter (arrow). (**B**) Southwestern blot analysis was performed using synthetic wild-type and −475 mutant oligonucleotides (−463 to −487, 25 bp). The loading control was stained with *ethidium bromide*. The Southwestern blot was blotted with HDGF synthesized by *in vitro* translation followed by an anti-HDGF antibody. The Western blot for HDGF synthesized by *in vitro* translation is shown. Positive signals of Southwestern blotting are indicated by black arrows. The wild-type oligonucleotide had a higher binding intensity than the −475 mutant oligonucleotides. (**C**) The Western blotting results for HDGF and OAT1 are shown. HK2 cells over-expressing HDGF were used as the positive control. Cells transfected with empty vector and those cultured under normal conditions were used as negative controls. The results indicate that over-expression of HDF down-regulated OAT1 expression in HK2 cells. The relative ratios *vs*. vector control after normalization with actin are plotted.

Hepatoma-derived growth factor (HDGF) is known as a transcription repressor^16,17^. LC/MS/MS results showed that the −475 mutant oligonucleotide significantly down-regulated HDGF binding (Table 3). Alignment analysis of the HDGF binding site of the SMYD1 promoter (30 nt) with the 5′ regulatory region of OAT1 (−463 to −487, 25 nt) revealed 43.3% sequence identity over a 30 nt overlap. In addition, the nucleotide at the −475 position of OAT1 was conserved in the HDGF binding site on the SMYD1 promoter (Fig. 4A)^17^. Southwestern blot analysis with HDGF produced by *in vitro* translation and synthetic wild-type and −475 mutant oligonucleotides (−463 to −487, 25 bp) showed that the −475 mutant oligonucleotide significantly down-regulated HDGF binding (Fig. 4B). To define the regulatory effects of HDGF on OAT1 expression, expression of OAT1 by HK2 cells over-expressing HDGF was analyzed by Western blot. Compared with control cells, cells over-expressing HDGF had significantly decreased OAT1 expression (Fig. 4C). These results suggest that the −475 SNP of OAT1 might down-regulate HDGF binding, resulting in over-expression of OAT1.

**Figure 5:**
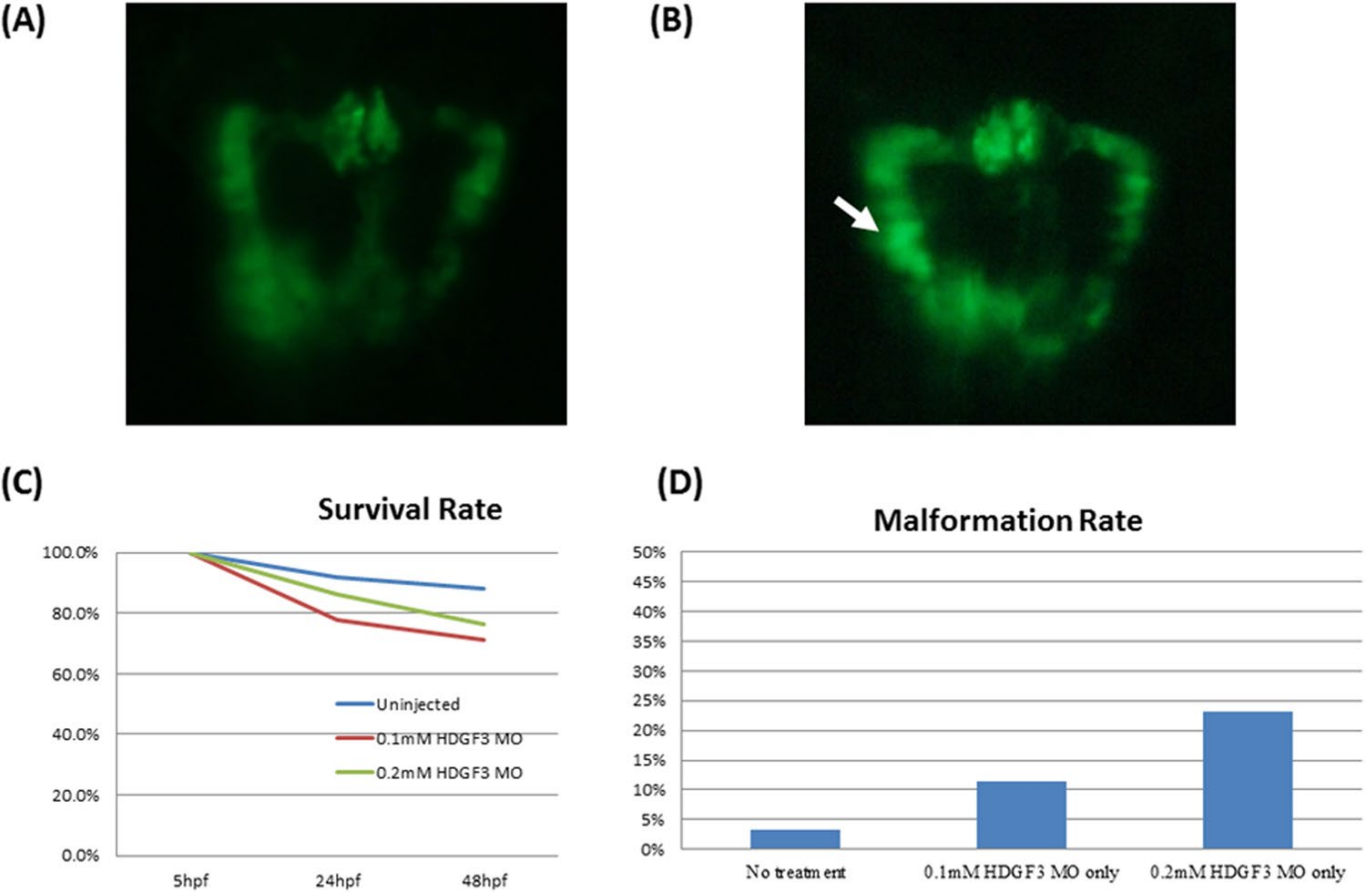
HDGF3 and kidney development in a zebrafish model. (**A**,**B**) The phenotypes of zebrafish kidneys with and without *HDGF3* morpholino antisense oligonucleotide injection were investigated (n = 30 for each group). At 48 hpf, the zebrafish kidneys were viewed via fluorescent microscopy and the deformity rates were analyzed. (**C**,**D**) The kidney deformity rates of zebrafish with and without *HDGF3* morpholino antisense oligonucleotide injection were 23.08% *vs*. 3.33% (Fisher’s exact test: P < 0.001).

To define the association of HDGF with kidney malformation, HDGF-deficient zebrafish embryos were obtained by injection of antisense morpholino oligonucleotides. The zebrafish embryos were produced from the green fluorescent kidney transgenic zebrafish line *Tg*(*wt1b:egfp*), which enables easier observation of kidney malformations. The results showed that embryos derived from *HDGF3*-MO injection displayed more malformed kidney phenotypes at 48 hpf than did embryos of the uninjected control group (defect rate 23.1% *vs*. 3.3%, n = 30; Fig. 5C,D). Differences in defects in the glomerulus, pronephric tube, and pronephric duct were observed between the uninjected control and *HDGF3*-MO-injected groups, particularly in fish with severe defects (Fig. 5A,B). These results indicate that *HDGF3* expression is essential for kidney development.

## Discussion

The central role of the kidney in the elimination of potential internal or external toxins from the blood into the urine is well documented. Substrates for OAT1 are varied and range from the classic small organic anion *para*-aminohippurate to several clinically important drugs, herbicides, and endogenous substances. For these reasons, there has been much interest in the possibility that polymorphisms in *SLC22A6* may be partially responsible for variation in the handling and efficacy of many commonly used drugs and toxins that are transported by OAT1^14,18,19^.

Substantial evidence indicates that OAT1 activity is critical in renal function and injury. A key role for OAT1 in the handling of uremic toxins derived from the gut microbiome was identified using *Oat1-*knockout mice^20^. The results of studies in *Oat1*-knockout mice suggest that uremic toxins and solutes are significantly retained in *Oat1*-knockout mice. On the other hand, OAT1 activity also has important roles in the pathogenesis of drug-related kidney injury. *In vivo* studies with *Oat1*-knockout mice verified that disruption of OAT1 activity can prevent renal toxicity of drugs or chemicals^21,22^. In *Oat1*-knockout mice, the loss of function of OAT1 was associated with decreased renal accumulation of arachidonic acid and lessened the severity of renal injury compared to wild-type animals^8^.

**Figure 6:**
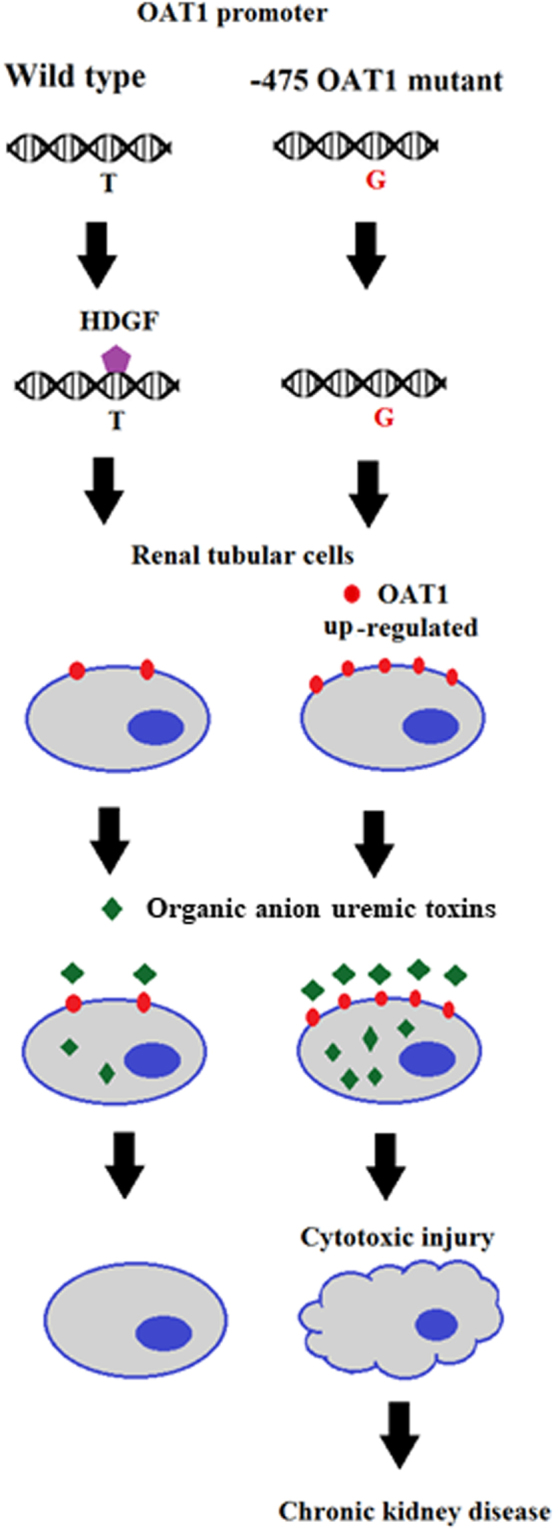
Mechanism of expression regulated by the −475 regulatory polymorphism of OAT1 in chronic kidney injury. The −475 regulatory polymorphism of OAT1 diminished the binding of HDGF, which functioned as a transcription repressor. Renal tubular cells with the −475 regulatory polymorphism had increased OAT1 expression, which resulted in increased transport of organic anion uremic toxins into cells. Cellular accumulation of organic anion uremic toxins caused cytotoxicity and resulted in kidney injury.

In a previous study with an ethnically diverse sample of 96 individuals, only one polymorphism was found in the 5′ regulatory region of OAT1^19^. In our study population (n = 324), there were 10 SNPs. Furthermore, our study also indicated that polymorphisms in the 5′ regulatory region of human OAT1 had significant clinical associations with CKD. Our results suggest that subjects with the −475 SNP (T > T/G) of OAT1 have increased risk of CKD. Our study also found that the −475 SNP with T to G transversion could increase OAT1 promoter activity that might result in increased OAT1 expression. OAT1 plays a major role in the renal uptake of uremic toxins on the basolateral membrane of renal tubules. Previous studies have indicated that OAT1 expression is associated with intracellular accumulation of organic anion toxins in the renal tubular cells of patients with CKD^7^. It has also been shown that transporter molecules such as OAT1 transport anionic uremic toxins into cells, where they accumulate and can cause oxidative stress and ultimately kidney injury^23–25^. Our results suggest that increased OAT1 expression due to the −475 SNP with T to G transversion might increase intracellular organic anion uremic toxins accumulation such that it exceeds the excretion rate, resulting in nephrotoxicity (Fig. 6).

Another important finding of our study was that the −475 SNP of OAT1 with T to G transversion could decrease HDGF binding. HDGF is considered a multi-functional protein and is suggested to have important roles in organ development^26^. HDGF shows proliferative activity, and expression of HDGF has been reported in many different tumor types and correlated with prognosis^27,28^. HDGF is also known as a transcription repressor. A microarray study with mouse primary aortic vascular smooth muscle cells demonstrated that expression of HDGF significantly down-regulated a large group of genes and increased expression of a relatively small number of genes^17^. Similarly, our study demonstrated that over-expression of HDGF could down-regulate OAT1 expression in cultured kidney cells, indicating that HDGF might be a transcription repressor for OAT1. The findings above suggest that the −475 SNP of OAT1 with T to G transversion might increase OAT1 expression by down-regulating HDGF binding to the OAT1 promoter thus lessening the transcriptional repression of OAT1.

In the present study, we identified a clinical association between SNPs of OAT1 and CKD. Our study demonstrated that SNPs of OAT1 can alter the transcriptional regulation of OAT1, which might affect CKD outcomes. Despite the low PCR error rate, this study may have been confounded by PCR errors that may have caused false SNP signals. Collectively, our data provide the first evidence of the clinical significance of SNPs of OAT1 on CKD and suggest that testing SNPs of OAT1 might serve as a valuable tool for CKD prevention and therapy.

## Methods

### Study subjects

A case-control study was conducted with sex- and age-matched groups. The inclusion criterion was adults aged >18 but <80 years. Patients were excluded from the study if they had diabetes mellitus, autoimmune disease, malignant disease, polycystic kidney disease, organ transplantation, infections requiring admission to the hospital in the past 3 months or an unwillingness to participate in the study. In total, 162 normal subjects and 162 subjects with CKD (eGFR < 30 ml/min/1.73 m^2^) were recruited into the study. This study adhered to the Declaration of Helsinki and was approved by the Ethics Committee of the Institutional Review Board at Chang Gung Memorial Hospital (Approval No. 103-0344C). Informed consent for all participants (162 normal subjects and 162 subjects with CKD) was obtained and kept at the Chang Gung Memorial Hospital.

### Leukocyte chromosomal DNA preparation

In brief, leukocytes were separated from a specimen of whole human blood by mixing the specimen with a hypotonic EDTA solution (1 mM). White blood cells were separated by centrifugation. The chromosomal DNA was extracted with an automatic nucleic acid extraction system according to the product instructions (LabTurbo 96 Standard System, Taigen Bioscience Corporation, Taipei, Taiwan).

### Chromosomal DNA sequencing and polymorphism identification

The 5′ regulatory region of OAT1 (−1196 to +88 relative to the transcription start site) was amplified by polymerase chain reaction (PCR) with chromosomal DNA. PCR was performed in 25 µL SYBR Green PCR Master Mix (Applied Biosystems, Waltham, MA) containing 0.6 mol/L primers (Table 4) and 1 µg DNA using an iQ5 PCR detection system (Bio-Rad, Berkeley, CA). Then, the PCR products (500 ng) were identified and purified by gel electrophoresis and sequenced with a capillary automatic DNA sequencing machine (ABI 3730, Thermo Fisher Scientific, Waltham, MA). Five primers (Table 4) were designed to complete sequencing of the 5′ regulatory region of OAT1. The sequencing results were analyzed by Sequencing Analysis software (v.5.3; Applied Biosystems, Waltham, MA). The 5′ regulatory polymorphisms of OAT1 were analyzed with Lasergene v7.2 software (DNASTAR, Madison, Wisconsin). The sequences of NT-167190.1 from the NCBI databank were used as the reference sequences. The SNP locations on chromosome 11 were obtained from the Ensembl database (http://asia.ensembl.org/index.html). The rs numbers of the polymorphisms detected by sequencing were obtained from the dbSNP database (https://www.ncbi.nlm.nih.gov/snp)^29^.

**Table 4:** Lists of primers.

OAT1 PCR	
FP1 (forward)	CAAGGCTGCAGTGTGCCAAGATTGT
FP3 (reverse)	TCCCTTGCAGCTTCTCCTCACTTTG
**OAT1 promoter**	
FP1 (forward)	CAAGGCTGCAGTGTGCCAAGATTGT
FP2S(forward)	AGACACTATGGACAGAAGACAAT
FP3S (forward)	GGGCACCCTGTAATTTCCCTGGCAA
RP1S(reverse)	CGTCATACAATGTCGGGTGATTC
1013R(reverse)	GGGATCTATTGGACCTATTTGT
**Luciferase**	
5′ primer	AGACGCCAA AAACATAAAGAAAGGCCCGGC
3′ primer	TATAAATGTCGTTCGCGGGCGCAACTGCAA
**Human 18S rRNA**	
5′ primer	CTACCACATCCAAGGAAGCA
3′ primer	TTTTTCGTCACTACCTCCCCG

### Sequence alignment

Multiple protein and DNA sequence alignments were performed using MAFFT (Multiple Alignment using Fast Fourier Transform) with the default parameter settings (http://www.ebi.ac.uk/Tools/msa/mafft/).

### Promoter activity assay

Wild-type and −475 mutant (T > G) oligonucleotides (−1 to −1196 of OAT1) were artificially synthesized and cloned into the polycloning sites (Xhol and Hind III) of the pGL4.17[luc2/Neo] vector (Promega, Fitchburg, Wisconsin). The wild-type and −475 mutant constructs were verified by sequencing (Supplemental Fig. S2). The luciferase activity of cell lysates was measured by the Luciferase Assay System (Promega) according to the product instructions and analyzed by a luminometer (Microplate Luminometer, Promega, Fitchburg, Wisconsin) (delay time: 2 seconds; read time: 10 seconds). Luciferase mRNA was quantified by real-time PCR with the primers listed in Table 4.

### Cell culture and transfection

HK2 cells were obtained from ATCC and cultured as suggested by ATCC. Cultured cells at ∼70% confluence were transfected with plasmids using Lipofectamine (1:1 ratio DNA to Lipofectamine) (Thermo Fisher Scientific, Waltham, MA). For HDGF over-expression, the cells were transfected with an HDGF open reading frame-containing plasmid (Lenti ORF clone of human HDGF, transcript variant 1, Myc-DDK-tagged; OriGene Technologies, Inc., Rockville, MD) The transfected cells were harvested and analyzed 48 hours after transfection.

### Chromatin immunoprecipitation and LC/MS/MS analysis

The nuclear extract of HK2 cells (1 × 10^6^ cells) was prepared with nuclear extraction reagents (NE-PER Nuclear and Cytoplasmic Extraction Reagents, Thermo Fisher Scientific) and incubated with biotin-labeled synthetic oligonucleotides (−463 to −487) with or without the −475 mutation (T to G). Chromatin immunoprecipitation was performed using an anti-biotin antibody (Abcam, Cambridge, Massachusetts). The nuclear protein extract was analyzed by 12.5% SDS-PAGE. After electrophoresis, the gels were stained with VisPRO 5 minutes Protein Stain kit (Visual Protein, Taiwan). After staining, the gels were washed in Milli-Q water and stored at 4 °C until processing for in-gel digestion. The gel lanes corresponding to the sample were cut into 5 slices, and each slice was processed for in-gel digestion according to the Shevchenko method. Briefly, each slice was washed/dehydrated three times in 50 mM ammonium bicarbonate (ABC, pH 7.9)/50 mM ABC +50% acetonitrile (ACN). Subsequently, cysteine bonds were reduced by incubating slices in 10 mM dithiothreitol for 1 h at 56 °C and alkylated by incubating slices in 50 mM iodoacetamide for 45 min at room temperature (RT) in the dark. After two subsequent wash/dehydration cycles, the slices were dried for 10 min in a vacuum centrifuge (ThermoFisher, Breda, The Netherlands) and incubated overnight with 6.25 ng/μL trypsin in 50 mM ABC at 25 °C. Peptides were extracted into 100 μL of 1% formic acid and then extracted twice into 100 μL of 50% ACN in 5% formic acid. The volume was reduced to 50 μL in a vacuum centrifuge before LC-MS/MS analysis. Peptides were separated using an Ultimate 3000 nanoLC system (Dionex LC Packings, Amsterdam, The Netherlands) equipped with a 20 cm × 75 μm i.d. fused silica column custom packed with 3 μm 120 Å ReproSil Pur C18 aqua (Dr. Maisch, GMBH, Ammerbuch-Entringen, Germany). After injection, peptides were trapped at 30 μL/min on a 5 mm × 300 μm i.d. Pepmap C18 cartridge (Dionex LC Packings, Amsterdam, The Netherlands) in 2% buffer B (buffer A, 0.05% formic acid in MQ; buffer B, 80% ACN and 0.05% formic acid in MQ) and separated at 300 nL/min in a 10–40% buffer B gradient over 60 min. Eluting peptides were ionized at 1.7 kV in a Nanomate Triversa Chip-based nanospray source using a Triversa LC coupler (Advion, Ithaca, NJ). Intact peptide mass spectra and fragmentation spectra were acquired on a LTQ FT hybrid mass spectrometer (Thermo Fisher). Intact masses were measured at a resolution of 50 000 in the ICR cell using a target value of 1 × 10^6^ charges. In parallel, following an FT prescan, the top 5 peptide signals (charge-states 2+ and higher) were submitted to MS/MS in the linear ion trap (3 amu isolation width, 30 ms activation, 35% normalized activation energy, Q-value of 0.25 and a threshold of 5000 counts). Dynamic exclusion was applied with a repeat count of 1 and an exclusion time of 30 s. MS/MS spectra were searched against the Homo sapiens SwissProt 2013_05 database (540,052 sequences; 191,770,152 residues) using Sequest (version 27, rev 12), which is part of the BioWorks 3.3 data analysis package (Thermo Fisher). MS/MS spectra were searched with a maximum allowed deviation of 10 ppm for the precursor mass and 1 amu for fragment masses. Methionine oxidation and cysteine carboxamidomethylation were allowed as variable modifications. Two missed cleavages were allowed, and the minimum number of tryptic termini was 1. After database searching, the DTA and OUT files were imported into Scaffold (versions 1.07 and 2.01) (Proteome Software, Portland, OR). Scaffold was used to organize the data and to validate peptide identifications using the Peptide Prophet algorithm. Only identifications with a probability >95% were retained. Subsequently, the Protein Prophet algorithm was applied, and protein identifications with a probability >99% with 1 or 2 peptides in at least one of the samples were retained. The LC/MS/MS data were analyzed by DAVID functional annotation tools (http://david.abcc.ncifcrf.gov/tools.jsp) and Metacore 6.1 software (GeneGo pathways analysis) (http://www.genego.com).

### Western and Southwestern blotting

Total protein was extracted using a commercial kit according to the manufacturer’s instructions (Protein Extraction Kit, Millipore, Billerica, Massachusetts). Then, 30 μg of protein from each sample was mixed with sample-loading buffer and loaded onto separate lanes of a 12% sodium dodecyl sulfate-polyacrylamide gel. The proteins were electrotransferred onto polyvinylidene fluoride membranes (0.2 μm: Immun-Blot, Bio-Rad) and then immunoblotted with antibodies against HDGF (Abcam), OAT1 (Abcam), and β-actin (Abcam). The intensity of each band was quantified using NIH Image software (Bethesda, Maryland), and the densitometric intensity corresponding to each band was normalized against β-actin expression.

HDGF protein for Southwestern blotting was synthesized using purified HDGF mRNA with a eukaryotic cell-free protein expression system (TnT® SP6 High-Yield Wheat Germ Protein Expression System; Promega). The HDGF mRNA for *in vitro* translation was obtained by *in vitro* transcription (HeLaScribe® Nuclear Extract *in vitro* Transcription Grade, Promega) using an HDGF open reading frame-containing plasmid (Lenti ORF clone of human HDGF, transcript variant 1, Myc-DDK-tagged; OriGene Technologies, Inc.). The *in vitro* translation product was verified as HDGF by Western blotting and used for Southwestern blotting analysis. The synthetic wild-type and −475 mutant oligonucleotides (−463 to −487, 25 bp) (20 μg) were transferred to nitrocellulose paper after electrophoresis with 3% ultra-pure agarose gel (Sigma-Aldrich Co. St. Louis, Missouri). After washing with PBS buffer, the transferred membrane was hybridized with HDGF protein and then immunoblotted with antibodies against HDGF (Abcam).

### Fish embryo staging and morpholino injection

Mature Tg(wt1b:EGFP)^30^ zebrafish were maintained at 28 °C with a photoperiod of 14-h light and 10-h dark in an aquarium supplied with freshwater and aeration. Embryos were produced using standard procedures^31^ and were staged according to standard criteria (hours postfertilization, hpf)^32^ or by days postfertilization (dpf). Antisense morpholino oligonucleotides targeting the 5′ untranslated region and the translation initiation site of HDGF *(HDGF3ATG-*MO: 5′-GGCGAGCCATGCCGACACAC-3′) were designed and obtained from Gene Tools (Philomath, OR). MOs were dissolved in 1× Danieau solution containing 0.5% Phenol red, and 2.3 nl of MO solution of the indicated concentration was injected into 1-cell-stage Tg(*wt1b:egfp*) embryos. All of the embryos were observed under a microscope (DM 2500, Leica, Wetzlar, Germany) equipped with a GFP fluorescent module. Pictures of the embryos were captured at particular stages with a digital camera (SONY, Tokyo, Japan).

### Statistical analyses

Descriptive statistics were expressed as the means ± standard deviation or percentage frequency, as appropriate. Paired *t*-tests were used to compare the means of continuous variables. Fisher’s exact test was used to analyze categorical variances in study subjects. The odds ratio was calculated with Pearson’s chi-squared test. A *P*-value < 0.05 was considered significant (two-tailed). One-way analysis of variance with Bonferroni corrections was used to analyze the data of the cell culture study. Data were analyzed using the commercially available SPSS 16.0 statistical software program (SPSS, Chicago, IL).

## Electronic supplementary material

Supplementary Figures
