# Supplementary material for: A novel SNP in the 5’ regulatory region of organic anion transporter 1 is associated with chronic kidney disease
Source: Sci Rep. 2018 May 24;8:8085. doi: 10.1038/s41598-018-26460-y (PMC5967335; doi:10.1038/s41598-018-26460-y)

A novel SNP in the 5' regulatory region of organic anion transporter 1 is associated with chronic kidney disease

Chiao-Yin Sun*[1, 2], Mai-Szu Wu[3, 4], Chin-Chan Lee[1, 2], Shu-Hong Chen[1, 5] , Kang-Chieh Lo[6], Yau-Hung Chen*[6]

1 Department of Nephrology, Keelung Chang Gung Memorial Hospital, Keelung, Taiwan;
2 College of Medicine, Chang Gung University, Taoyuan, Taiwan;
3 Department of Internal Medicine, School of Medicine, College of Medicine, Taipei Medical University, Taipei, Taiwan;
4 Division of Nephrology, Department of Internal Medicine, Taipei Medical University Hospital, Taipei, Taiwan;
5 Medical Research Center, Keelung Chang Gung Memorial Hospital, Keelung, Taiwan;
6 Department of Chemistry, Tamkang University, Tamsui, New Taipei City, Taiwan.

*Correspondence and requests for materials should be addressed to Chiao-Yin Sun (email: fish3970@gmail.com) or Yau-Hung Chen (email: yauhung@mail.tku.edu.tw)

Running title: Organic anion transporter 1 and chronic kidney disease

Suppl figure 1: The reference sequence of 5’ regulatory region of OAT1 and promoter prediction. (A) The reference sequence of 5’ regulatory region of human OAT1 was from the genomic data of Ensembl website (http://asia.ensembl.org/index.html).

(B) The sites of TATA and CCAAT boxes were predicted with GPMiner (http://gpminer.mbc.nctu.edu.tw/index.php). **(C)** The promoter prediction was analyzed by NNPP version 2.2 with default setting (http://www.fruitfly.org/seq_tools/promoter.html).

A

CAAGGCTGCAGTGTGCCAAGATTGTGCCACTACACTCCAGGCTGGATGACAGAGTGAGACCCTATCTCAAAAATAAATTA
AAGAGGCCAGGCATGGTGTCTCACGCCTATAATCCCAGCACTTTGGGAGGCTGAGATGGGCAGATCACCTGAAGTCAGG
AGTTCGAGACCAGCCTGGCCAACATGGTGACACCCCATCTCTACTAAAAATAACAAAAATTAGCCAGGCGTGGTAGCACAT
GCCCATAATCCCAGCTACTCGGGAGGTTGAGGCATGATAATCACTTGAACCTGGGAGGCAGAGGTTGCAGTGAGCTGAGA
TTGCACCATTGCACTCCAGCCTGGGCAACAGAGTGAGACTGTGTCTCAAAAAGAAAGAGAAGAGAGGAGAAGAGAAGA
GAAGAGGGGAGGAGAGGGGAGGGGAGCGAAGGGGAGGTGACAAAGGAGGGGAGGGGAGGGGAGAAGAGAAGAA
AACAAGAAGAAAAGAAAAGAAAAAAGACACTATGGACAGAAGACAATATGACTAAGGGAAAAATCTGACCCTTGGAAG
GGTCAGAAGAATCACTGGAAGAATCACCCGACATTGTGTATGACGTTGGTCTTAGGTCCCACTCCAAGAGCGGCCTCTGA
CTTCTGTTGTTTCTTCCAAATGAATAAAACCATGGTTCTATAAGATGTGAGCTGTCCCCAGAAGAACAAAGACTGGGGTTGT
CACATTTAGCAAATAAAGATACAGGGTGCCCAGTCAAATTTGCATTTCAAATCAATGAAGAATTGCTTAGTATAAGTATGTCT
CATGCAATATTAGAGACATACTCATACTTTTAAAAACTCTTCATTGACCTAAAATGCAAATTTGACTGGGCACCCTGTAATTTC
CCTGGCAACCCTTCCCAAAAGCAGACTATTTGGATCTCATCCACTAAAAAGGGCAGAAACTCCTCAAGAGGCCACCACAC
TTCCCAGAGCCCAGTCTCCTTCCTAAAAGGAAGACAAATAGGTCCAATAGATCCCACTCTGGCCCCCCCTGCCCCCAGATG
CCCCCCTAATACACCCCTCTCCCTGCTCCTATTCAGTCCACCCTCTCCTGCCCTTTATAACCACTTGGAGAAATTCCACTGACA
CAAGGAATCCTTGGAGGGTTAATCCTTCTGATACCAAGTCACACTTTAACTCATTCCCTCCAGGCCAAGGATTAAAAACTG
CCCATGCAAGGGTCAGGTCTCCAGCAGACCCTGAAAGCTGAGCTGCCCTGACCCCCAAAGTGAGGAGAAGCTGCAAG
GGAA

ATG : translation starting site **A: transcription starting site** **T : -1** **C : -1196**

B

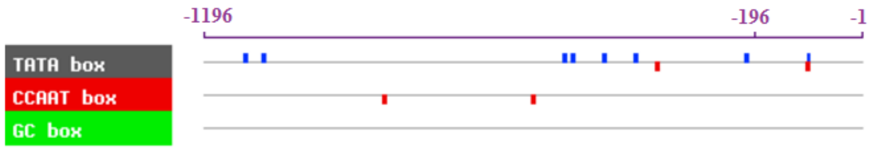

C

Start	End	Score	Promoter Sequence
-1130	-1080	0.96	TCAAAAATAAATTAAAGAGGCCAGGCATGGTGTCTCACGCCTATAATCCC
-539	-489	0.98	ACCATGGTTCTATAAGATGTGAGCTGTCCCCAGAAGAACAAAGACTGGGG
-285	-235	0.99	ATCTCATCCACTAAAAAGGGCAGAAACTCCTCAAGAGGCCACCACACTTC

Suppl figure 2: Sequencing verification for wild type and -475 mutant OAT1 promoter constructs.

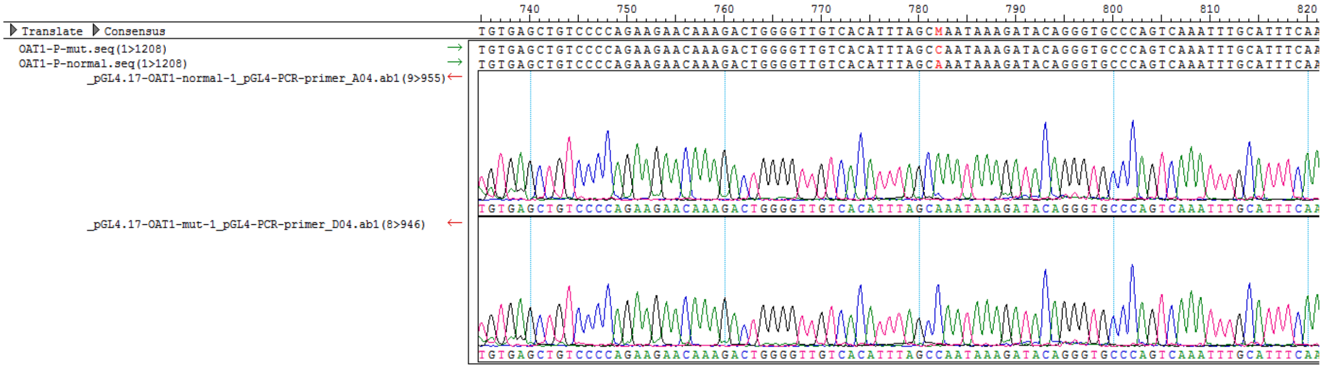

Supplement: Supplementary file 1 — Supplementary Figures [file 41598_2018_26460_MOESM1_ESM.pdf]
